# Membrane Lipid Co-Aggregation with α-Synuclein Fibrils

**DOI:** 10.1371/journal.pone.0077235

**Published:** 2013-10-11

**Authors:** Erik Hellstrand, Agnieszka Nowacka, Daniel Topgaard, Sara Linse, Emma Sparr

**Affiliations:** 1 Division of Biophysical Chemistry, Center of Chemistry and Chemical Engineering, Lund University, Lund, Sweden; 2 Division of Physical Chemistry, Center of Chemistry and Chemical Engineering, Lund University, Lund, Sweden; 3 Division of Biochemistry, Center of Chemistry and Chemical Engineering, Lund University, Lund, Sweden; University of Pittsburgh School of Medicine, United States of America

## Abstract

Amyloid deposits from several human diseases have been found to contain membrane lipids. Co-aggregation of lipids and amyloid proteins in amyloid aggregates, and the related extraction of lipids from cellular membranes, can influence structure and function in both the membrane and the formed amyloid deposit. Co-aggregation can therefore have important implications for the pathological consequences of amyloid formation. Still, very little is known about the mechanism behind co-aggregation and molecular structure in the formed aggregates. To address this, we study in vitro co-aggregation by incubating phospholipid model membranes with the Parkinson’s disease-associated protein, α-synuclein, in monomeric form. After aggregation, we find spontaneous uptake of phospholipids from anionic model membranes into the amyloid fibrils. Phospholipid quantification, polarization transfer solid-state NMR and cryo-TEM together reveal co-aggregation of phospholipids and α-synuclein in a saturable manner with a strong dependence on lipid composition. At low lipid to protein ratios, there is a close association of phospholipids to the fibril structure, which is apparent from reduced phospholipid mobility and morphological changes in fibril bundling. At higher lipid to protein ratios, additional vesicles adsorb along the fibrils. While interactions between lipids and amyloid-protein are generally discussed within the perspective of different protein species adsorbing to and perturbing the lipid membrane, the current work reveals amyloid formation in the presence of lipids as a co-aggregation process. The interaction leads to the formation of lipid-protein co-aggregates with distinct structure, dynamics and morphology compared to assemblies formed by either lipid or protein alone.

## Introduction

Amyloid deposits from several human diseases have been found to contain membrane lipids [1], [2]. In Parkinson's disease, the amyloid forming protein α-synuclein is deposited together with lipids in the core of brainstem Lewy Bodies (LB), and lipids are also diffusively distributed in cortical LB. The fibrillar LB aggregates, which are the neuropathological hallmarks of the disease, also contain other protein components, of which many are membrane associated [3]–[5]. The presence of membrane components in the formed amyloid deposits implies an uptake of these components into the aggregates during or after the formation process. Co-aggregation is expected to have large consequences for the physico-chemical properties of the formed aggregates and modulate their interactions. It also implies extraction of components from the membrane, which likely affects the membrane structure and function. Co-aggregation of amyloid proteins and membrane components can therefore have pathological consequences.

**Figure 1:**
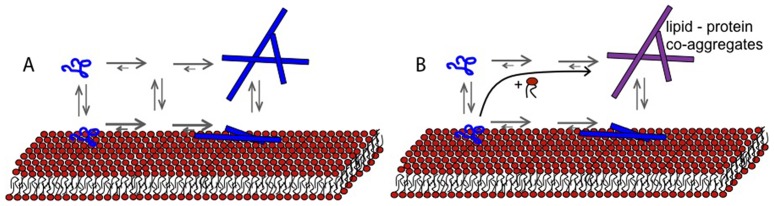
Schematic illustration of amyloid fibril formation in the presence of a lipid membrane. In the majority of published studies, the processes illustrated in (A) are investigated, including studies of the aggregation process (monomeric protein, via transient oligomeric aggregates to amyloid fibrils), and studies of how proteins in different aggregation stages adsorbs to and modify lipid membranes. In the present study, we focus on another aspect of the amyloid formation process, that is co-aggregation and the possibility of lipid uptake from the membrane into the fibrillar aggregates (B).

Protein aggregation *in vivo* is governed by intrinsic (amino acid sequence and covalent modifications) as well as extrinsic factors, including a crowded environment and the presence of surfaces and a large number of molecular species of different size and hydrophobicity. For several of the amyloid diseases, protein aggregation has been associated with membrane disruption in cells and in model lipid systems [6]–[9]. In particular, there is growing evidence that lipids play an important role in the pathology of Parkinson’s disease [2], [8], [10]. There are numerous reports showing protein adsorption to lipid membranes, and that the lipid membranes interfere with the aggregation process. In addition, protein in different aggregation states (monomeric, oligomeric or fibrillar) can lead to alterations in membrane morphology and permeability [11]. Most of these studies focus on the interaction of the protein with the (intact) lipid membrane, as illustrated in (Figure 1a).

In the present study, we take a different approach and study the uptake of lipids into the forming amyloid aggregates (Figure 1b), as this is a potentially important factor to the understanding of amyloid fibril formation *in vivo* and its effects on cells. We explore co-aggregation of phospholipids and the amyloid protein α-synuclein. A key element in this approach is the view of the lipid membrane as a self-assembled dynamic structure rather than an intact and inert entity. The amphiphilic lipids can rearrange into new assemblies together with other macromolecules when the conditions are changed. Furthermore, aggregation is a dynamic process precluding isolation of on-pathway intermediate species. However, it is feasible to study the ongoing process and the end states as a function of the molecular composition or presence of surfaces during aggregation. Previous fluorescence confocal microscopy and surface sensitive fluorescence microscopy studies of protein aggregation in the presence of giant unilamellar vesicles have revealed extensive lipid-protein co-aggregation for different amyloid proteins, including α-synuclein [10], [12]–[15]. However, these studies do not provide any quantitative measures of the lipid content, or any characterization of the lipid structure in the deposits, and this forms the starting point for the present work.

We characterize the structure and composition of aggregates formed when the amyloid protein α-synuclein has been allowed to aggregate in the presence of phospholipid vesicles composed of zwitterionic and anionic lipids. The stoichiometry and specificity of the lipid uptake into the amyloid deposits is investigated using quantitative phosphorous analysis, the mobility of lipids in aggregates in comparison with unperturbed lipids in lamellar phase is explored using polarization transfer solid-state NMR (PT ssNMR) [16], and the morphology of aggregates and co-existing vesicles is studied by means of cryogenic transmission electron microscopy (cryo-TEM). All three techniques reveal co-aggregation of membrane lipids with α-synuclein in a saturable manner, and that the lipid uptake is sensitive to the composition of the model membrane. Moreover, there is reduced molecular mobility in specific regions of the phospholipid acyl chain when present in the amyloid deposit. Finally, we demonstrate distinct morphological changes in fibril aggregates when protein is co-aggregated with lipids.

## Materials and Methods

### Materials

All chemicals were of analytical grade and water was of Milli-Q grade. The phospholipids, 1,2-dioleoyl-sn-glycero-3-phospho-L-serine sodium salt (DOPS) and 1,2-dioleoyl-sn-glycero-3-phosphocholine (DOPC) were bought lyophilized from Avanti Polar Lipids (Alabaster AL) and were then used from stock solutions in CHCl3:MeOH 9∶1 stored at −20°C. Human α-synuclein was expressed in *Escherichia coli* from the aS-pT7-7 plasmid kindly provided by H. Lashuel as previously described [13].

### Sample Preparation

Monomeric α-synuclein was purified from lyophilized powder dissolved in 6 M guanidine HCl, pH 8 by size exclusion chromatography on a Superdex 75 column (GE Healthcare) into experimental buffer (20 mM MES pH 5.5, 0.02% NaN_3_). The monomer was then kept on ice until incubation with or without small unilamellar vesicles (SUVs). Protein samples without lipid were prepared identically but mixed with buffer instead of buffer solution with SUVs.

Lipid samples were prepared from 2–5 mM stock solutions of phospholipids in chloroform/methanol 9∶1 v/v. Thin lipid films were deposited onto glass under a slow flow of nitrogen and dried in vacuum overnight at room temperature. The films were hydrated with 20 mM MES/NaOH pH 5.5, 0.02% NaN_3_. Lipid samples for co-aggregation with protein were sonicated into small unilamellar vesicles (SUV) until clear solution with precautions taken not to overheat the samples. The SUVs were centrifuged to remove any traces of metal from the probe sonicator and were then incubated with 34 µM α-synuclein monomer overnight in plastic tubes with 200 rpm shaking at 37°C for maximum 20 h. The cryo-TEM samples were collected directly from the resulting solution while the aggregates were collected by centrifugation at 13 000×g for 5 minutes in the phosphorous quantification and NMR-measurements. For the control samples that only contain the lipid lamellar phase and no protein, the hydrated film was released from the glass by a couple of short bursts of sonication and was then pelleted by centrifugation and transferred into NMR rotor inserts.

### Lipid Quantification

**Figure 2:**
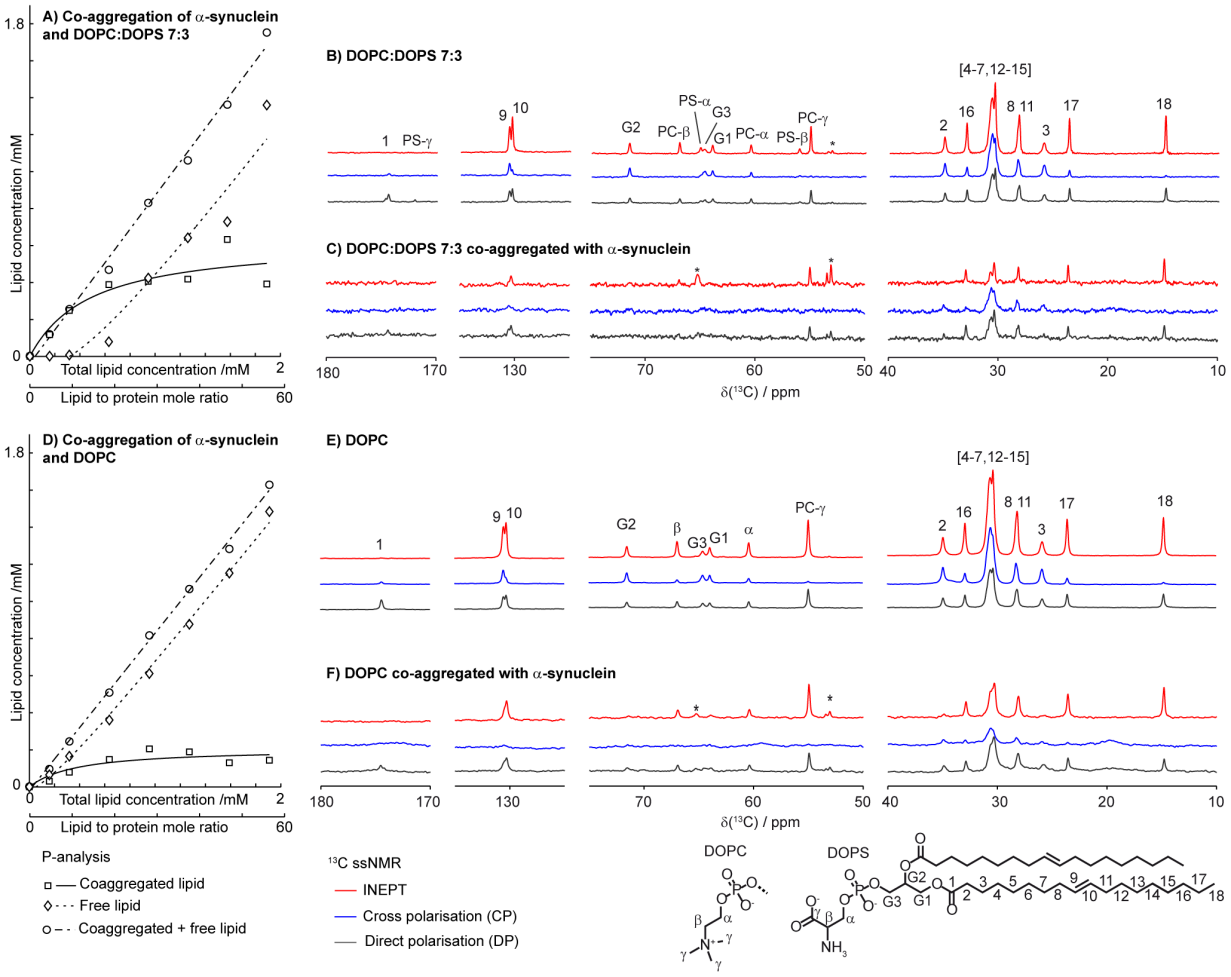
Co-aggregation of α-synuclein with DOPC:DOPS 7∶3 (a, c) and DOPC (d, f) at L/P = 18 (molar ratio). Left: Lipid concentration derived from quantitative phosphorous analysis of aggregated and non-aggregated lipids after co-aggregation with 34 µM α-synuclein (a, d). Right: Polarization transfer solid state NMR on lamellar phase lipids (b, e) and lipids co-aggregated with α-synuclein (c, f). Stars indicate peaks originating from buffer molecules. Spectra are normalized to equal intensity for DP of C_18_.

Phospholipid concentrations were determined by phosphate analysis according to Rouser et al [17]. Dried samples were digested with 0.65 ml 70% perchloric acid at 180°C for 20 minutes. When cool, 3.3 ml H_2_O, 0.5 ml 25 g/l ammonium molybdate and 0.5 ml 100 g/l ascorbic acid were added. After 5 minutes of incubation at 100°C, absorbance was measured at 800 nm. KH_2_PO_4_ was used to prepare a linear series of standard samples containing between 0 and 5 µg phosphorous. The experiments presented in Figure 2a and 2d were repeated twice with very similar results.

### Thin Layer Chromatography (TLC)

To analyze the composition of phospholipids in the co-aggregates and test for degradation of phospholipids, thin layer chromatography (TLC) was performed. Protein and phospholipids were incubated alone or together for 16 h at 37°C with shaking at 200 rpm. After centrifugation for 6 minutes at 13000×g, the fractions were lyophilized for three days and then dissolved in chloroform:methanol 2∶1. Samples (5–20 µl) were spotted onto aluminium-supported silica gel 60F254, which was developed in chloroform/methanol/water 65∶25:4 (by volume). Molybdenum blue spray reagent (Sigma-Aldrich, Sweden) was used for detection.

### Thioflavin T Test

To test if a sample contained amyloid fibrils, 2 mM Thioflavin T was added to a final concentration of 10 µM. The fluorescence when exciting at 440 nm was measured using a fluorescence spectrometer (Perkin Elmer, MA, USA). If a noticeable peak around 482 nm was observed, the sample was considered to be Thioflavin T positive indicating presence of fibrils. Control experiment with only buffer and Thioflavin T was done in parallel.

### Solid-state NMR

Solid-state NMR experiments were performed with a Bruker Avance-II 500 spectrometer (Bruker, Karlsruhe, Germany) using a 4 mm CP/MAS HX probe at the field of 11.7 T, resulting in ^1^H and ^13^C resonance frequencies of 500 and 125 MHz, respectively. The temperature was set to 25°C, using a BVT-2000 temperature control and cooling of the bearing air by a BCU-05 unit, with sample heating induced by MAS and radiofrequency pulses taken into account.

PT ssNMR ^13^C spectra were acquired using a spectral width of 250 ppm and an acquisition time of 50 ms, under 68 kHz TPPM ^1^H decoupling [18]. For each ^13^C spectrum, 2048 scans were accumulated with a recycle delay of 5 s, resulting in 2 h and 30 min of experimental time. The ^13^C chemical shift was externally referenced to solid α-glycine at 43.67 ppm [19]. ^1^H and ^13^C hard pulses were applied at *ω*_1_^H/C^/2*π* = 80 kHz. CP [20] was performed with *t*_CP_ = 1 ms, *ω*_1_^C^/2*π* = 80 kHz and *ω*_1_^H^/2*π* linearly ramped from 72 to 88 kHz, covering the ±*ω*_R_ matching conditions, and INEPT [21] with the delay times of *τ* = 1.8 ms and *τ*’ = 1.2 ms. Line broadening of 10 Hz, zero-filling from 1597 to 8192 time-domain points, Fourier transform, automatic phase correction [22], and baseline correction were used in processing the experimental time-domain data with a Matlab (www.mathworks.com) in-house code partially derived from matNMR [23]. Lipid peak assignment was made based on DOPC and DOPS spectra, confirmed by previous research [24], [25]. The experiments presented in Figure 2b and 2c were repeated twice with very similar results.

### Cryogenic Transmission Electron Microscopy (cryo-TEM)

Specimens for electron microscopy were prepared in a controlled environment vitrification system (CEVS) to ensure stable temperature and to avoid loss of solution during sample preparation. The specimens were prepared as thin liquid films, <300 nm thick, on lacey carbon filmed copper grids and plunged into liquid ethane at −180°C. This leads to vitrified specimens with preserved original microstructures, avoiding component segmentation and rearrangement, and water crystallization. The vitrified specimens were stored under liquid nitrogen until measured. An Oxford CT3500 cryoholder and its workstation were used to transfer the specimen into the electron microscope (Philips CM120 BioTWIN Cryo) equipped with a post-column energy filter (Gatan GIF100). The acceleration voltage was 120 kV. The images were recorded digitally with a CCD camera under low electron dose conditions.

## Results

### α-Synuclein co-aggregates with Lipids

We study the uptake of lipids into α-synuclein fibrillar aggregates. The experiments were designed so that monomeric recombinant α-synuclein freshly collected from size exclusion column was incubated together with small unilamellar vesicles (SUVs). The vesicles were composed of zwitterionic DOPC or a 7∶3 mixture of DOPC and anionic DOPS, chosen as a minimalistic model for intra-cellular membranes, exosomes and endosomes [26]–[28]. Over time, the vesicles spontaneously fuse to form larger aggregates of the equilibrium lamellar phase, but remain stable as unilamellar vesicles over the time frame of the experiment (up to 24 h), and they do not pellet upon centrifugation. However, when lipid vesicles are incubated together with α-synuclein, we find that the protein forms amyloid fibrils and co-aggregates with lipids from the vesicles. Protein aggregates were separated from unbound lipids by centrifugation, followed by quantitative phosphorous analysis of the pellet and supernatant, respectively. The results show lipid uptake into the protein aggregates, which is saturable for both model systems. However, there is a clear selectivity for the bilayer that contain anionic lipids with more than double lipid uptake in the protein fibril aggregates for DOPC:DOPS 7∶3 compared to purely zwitterionic DOPC bilayer (Figure 2, left panel). With the anionic lipid mixture, approximately 0.3–0.5 mM phospholipid is taken up into the protein deposits. With 34 µM α-synuclein, this corresponds to around 9–15 lipids per protein (L/P). Protein aggregation in the presence of DOPC vesicles results in phospholipid uptake of less than 0.2 mM, corresponding to L/P less than 6. The selective co-aggregation with lipids from vesicles that contain anionic DOPS does not necessarily mean that DOPS is enriched in the co-aggregates. Indeed, TLC separation of DOPS and DOPC from the co-aggregates, the supernatant and the reference sample without protein show similar lipid composition (Figure S1).

Lipid protein co-aggregation and sensitivity to membrane composition were studied in more detail using natural-abundance ^13^C polarization transfer solid state NMR (PT ssNMR). The PT ssNMR experiments give atomically resolved information per carbon in the phospholipid acyl chains in the neat lipid phase and in the co-aggregates. The ^13^C spectra in Figure 2 (right panel) clearly confirm the presence of lipids in the co-aggregated samples. The unresolved peaks in the ^13^C spectra around 20 ppm in Figure 2c and 2f originate from the protein. The samples were also thioflavin T positive indicating the presence of fibrillar protein. The spectra of the co-aggregated samples at these compositions are dominated by signals from the lipids which have higher intensities due to the higher concentration on molar basis.

In the following sections, we will focus on the characterization of the samples that contain anionic DOPS, as this system shows the highest lipid uptake into the co-aggregates. This will be followed by a comparison between the aggregates formed in the presence of mixed DOPC/DOPS model membranes and aggregates formed in the presence of purely zwitterionic DOPC model membranes.

### Decreased Lipid Dynamics upon Co-aggregation

The co-aggregated lipid-protein samples were studied by means of PT ssNMR, using three combined 1D ^13^C NMR experiments. The different NMR experiments were direct polarization (DP), and two experiments that act as mobility filters: cross polarization (CP) [20] and refocused insensitive nuclei enhanced by polarization transfer (refocused INEPT) [21]. Together these experiments give atomically resolved qualitative information on molecular dynamics in the different molecules [16], [29], [30]. For transfer of polarization from ^1^H to ^13^C nuclei, CP depends on through-space ^1^H-^13^C dipolar couplings, which are averaged to zero by rapid isotropic reorientation of the ^1^H-^13^C inter-nuclear vectors, making CP efficient for rigid molecules only. Therefore, only rigid molecules or molecular segments display high signal intensity in CP spectra. The INEPT sequence transfers polarization via the through-bond *J*-couplings, which are unaffected by bond reorientation. Furthermore, INEPT provides no signal from rigid molecules, due to fast *T*_2_ relaxation originating from the non-averaged ^1^H-^13^C dipolar couplings. The ratio between signal intensities in the CP and INEPT spectra thus depends on the reorientational dynamics of the C-H bonds. Processes such as bond vibration and trans-gauche isomerization, as well as rotational and translational diffusion of the entire molecule or aggregate, contribute to the C-H bond reorientation. For lipid bilayers, it is convenient to separate the types of reorientation into two classes: “fast” motion with an effective correlation time τ_c_ and order parameter *S*_CH_, and “slow” motion leading to completely isotropic reorientation. The slow mode corresponds to translational diffusion of the lipids between differently oriented bilayer patches or rotational diffusion of the entire vesicle, while the fast mode includes all other types of motion. The value of *S*_CH_ is given by the time-average of the expression (3cos^2^ *θ*−1)/2, where *θ* is the angle between the bilayer normal and the C-H bond vector, and the average is limited to times over which the molecules experience a constant bilayer orientation. Isotropic bond reorientation with respect to the bilayer normal, as well as preferential orientation at the angle *θ* = 54.7°, both lead to *S*_CH_ = 0.

Table 1 summarizes the different dynamic regimes that give rise to characteristic relative intensities in the PT ssNMR experiment [30]. With slow dynamics, τ_c_ >0.1 ms, CP dominates with no DP or INEPT signal. The lack of DP is due to slow ^13^C T_1_ relaxation. In the intermediate regime, τ_c_ ≈ 1 µs, both CP and INEPT are inefficient but DP begins to give visible signals. When increasing the motion to a correlation time of τ_c_ = 0.1 µs, i.e. to the fast-intermediate regime, CP and DP are efficient while INEPT is not. As an example, lipids in a liquid crystalline lamellar (L_α_) phase are in a fast regime with τ_c_ <1 ns. In this regime, DP is efficient, and the CP and INEPT efficiencies are not dependent on τ_c_ but solely depend on the order parameter |*S*_CH_|. A C-H bond with highly anisotropic reorientation, |*S*_CH_|>0.5, has an efficient CP but negligible INEPT. With a nearly isotropic bond vector reorientation, |*S*_CH_|<0.01, the opposite is true with an efficient INEPT and negligible CP, while at |*S*_CH_| ≈ 0.1, INEPT and CP are equally efficient. In a L_α_ palmitoyl chain, |*S*_CH_| is approximately 0.2 for C_2_–C_8_ and then smoothly decreases to zero over C_9_–C_16._ The same trend is seen for an oleoyl chain (C_2_–C_18_) but with a dip involving C_7_–C_11_ around the double bond with a minimum |*S*_CH_| ≈ 0 for C_10_ [24], [31].

**Table 1:** Dynamic regimes and resulting intensities from polarisation transfer solid-state NMR experiments [30].

Dynamic regime	τ_c_	|*S*_CH_|	Intensity
Fast	<1 ns	<0.01	INEPT>>CP = 0
		≈0.1	INEPT≈CP
		>0.5	CP>>INEPT = 0
Fast-intermediate	0.1 µs		CP≈DP>>INEPT = 0
Intermediate	1 µs		DP>CP = INEPT = 0,
Slow	>0.1 ms		CP>>DP = INEPT = 0

**Figure 3:**
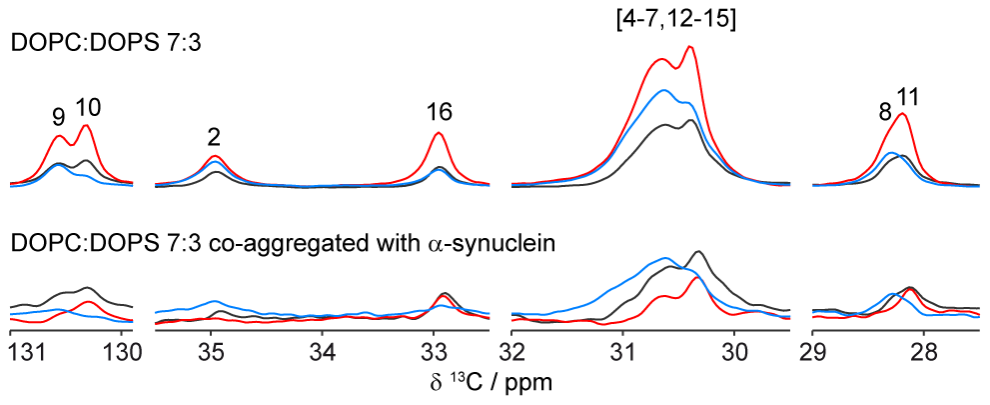
PT ssNMR spectra (DP black, CP blue, INEPT red) of DOPC:DOPS 7∶3 (top) and DOPC:DOPS 7∶3 co-aggregated with α-synuclein (bottom) at L/P = 18 (molar ratio). Numbers refer to assignments of the acyl chain from figure 2. Spectra are scaled to equal DP intensities at 30–31 ppm.

**Figure 4:**
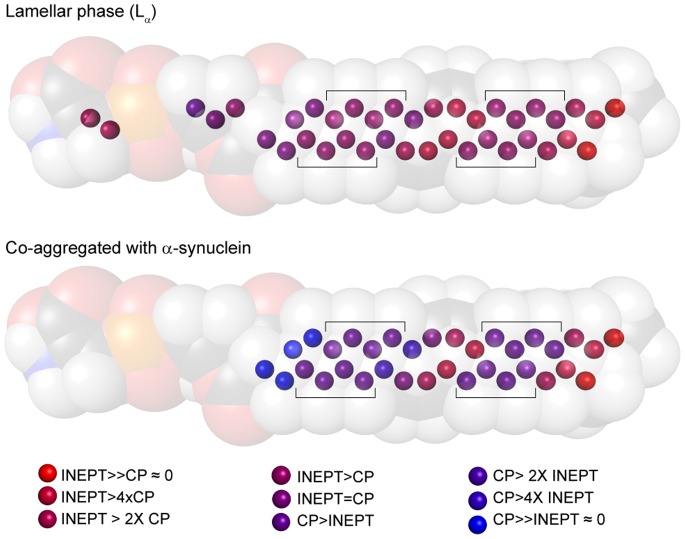
Relative intensities from INEPT and CP experiments for DOPC:DOPS 7∶3 in lamellar phase (top) and co-aggregated with α-synuclein (lipid/protein ratio, L/P, 1/1; bottom) (DOPS is used in representation). Brackets indicate the unresolved group of peaks from C_4–7_ together with C_12–15_. I_INEPT_/I_CP_ depends on both the correlation time τ_c_ and the order parameter |S_CH_| for the bond vector in the molecular segment. Detailed interpretation is given in Table S1.

The lipid-protein co-aggregated samples were investigated in parallel with two control samples that contain the lipid bulk lamellar phase or aggregated α-synuclein, respectively, and selected NMR data are shown in Figure 2 and 3. Based on the PT ssNMR experiments, we are now able to deduce atomically resolved information on the dynamics in different parts of the lipid molecules. The results obtained for the lipids present in the co-aggregates and in the neat lipid lamellar phase are summarized in the color-coded molecular structure in Figure 4.

The polarization experiments for the control lipids in Figure 2b, 2e show typical spectra for an anisotropic liquid-crystalline structure, such as the L_α_ lamellar phase, where the CP and INEPT efficiencies are determined by the value of |S_CH_| [30]. The measured intensities from the different polarization experiments are similar, although the INEPT signal is typically higher than the corresponding CP signal for most peaks from the lipid hydrocarbon chains, including the main chain peak at 30.5 ppm, which originates from the unresolved signals from C_4_–C_7_ and C_12_–C_15_. The absence of ^1^H in the C_1_ segment leads to the lack of information from that segment but C_2_ and C_3_ are showing higher |S_CH_| compared to the rest of the acyl chain. The end methyl C_18_ is the most isotropic molecular segment of the acyl chains due to free rotation around the C_17_–C_18_ bond. The corresponding data for lipids co-aggregated with protein are shown in Figure 2c, 2f. A closer inspection of the spectra reveals quantitative and significant changes in the relation between INEPT, CP and DP for several carbons in the lipids, and the most important regions of these spectra are shown enlarged in Figure 3. Thanks to the scarce protein signals in Figure 2c, 2f, we are able to resolve all peaks originating from lipid hydrocarbon chain and make quantitative comparisons between the spectra from lipids present in amyloid aggregates together with α-synuclein, and lipids present in a neat lamellar phase.

In the molecular structure shown in Figure 4, the observed ratios of INEPT and CP signal (*I*_INEPT_:*I*_CP_) for phospholipids present in lamellar phase or co-aggregated with α-synuclein are color coded (Table S1). Red corresponds to carbons for which INEPT dominates and blue to carbons for which CP dominates, relating to segments experiencing low or high |S_CH_| in the fast dynamic regime. From the comparison in Figure 4, it is clear that most segments in the lipid hydrocarbon chain give reduced INEPT signal after co-aggregation with α-synuclein. Thus the NMR data reveals that the mobility is affected for most of the carbons in the acyl chain after co-aggregation with α-synuclein compared to the lamellar phase. Looking closer at the double bond involving C_9_ and C_10_ between the unresolved CH_2_ segments (marked with brackets), it can be noticed that the change in *I*_INEPT_:*I*_CP_ ratio upon co-aggregation is very low for C_10_ and C_11_. Also the chain terminus, C_18_, and the adjacent C_17_, as well as the phosphatidylcholine C_γ_ (Table S1) are less affected by co-aggregation, although there is still a detectable decrease in *I*_INEPT_:*I*_DP_ ratio. It is possible that this small change is due in part to an increase in τ_c_ on the ns time scale. The peaks from other carbon atoms in the headgroup are within the level of noise due to the low lipid concentration in the co-aggregated samples.

### Co-aggregation Turns Fibril Bundles into Tangles

**Figure 5:**
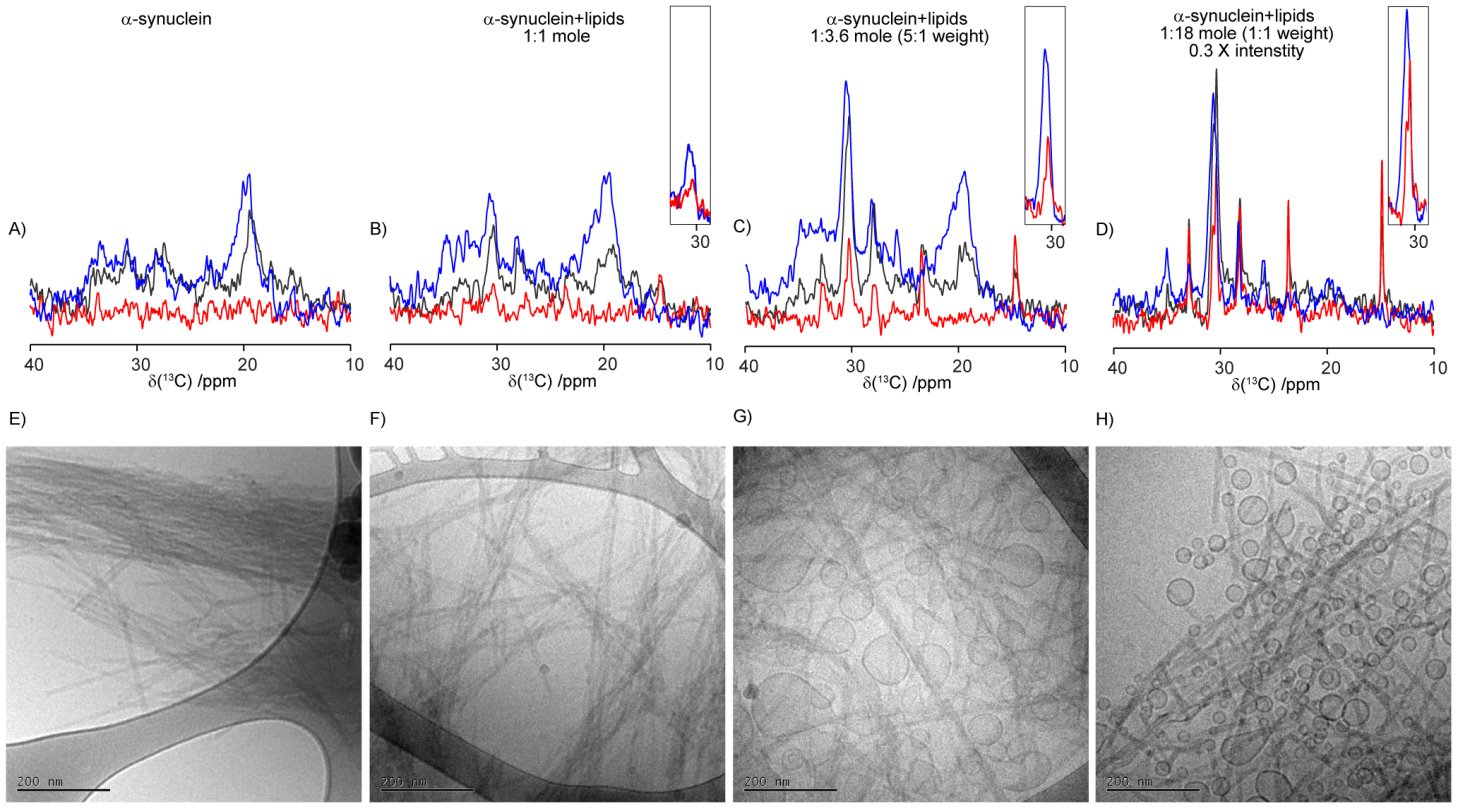
Main acyl chain region of the PT ssNMR spectra (a–d) and cryo-TEM images (e–h), scale bars 200 nm) of α-synuclein fibrils co-aggregated with different amounts of DOPC:DOPS 7∶3 vesicles. Inserted boxed peaks are INEPT and CP signals for the unresolved peaks from C_4–7_ and C_12–15_ baseline adjusted for background protein CP signal. Spectra are scaled to give equal protein CP intensity at 20 ppm. Filled dark spots in the cryo-TEM images are frost defects and are not part of the experimental system. More representative images can be found in Figure S3.

From the phosphate analyses presented in Figure 2, it was concluded that the lipid uptake into the aggregates reaches a level of saturation. To further investigate the molecular basis of this saturation, we studied protein-lipid aggregates formed in solutions with different L/P molar ratios by means of cryo-electron microscopy. Representative images are shown in Figure 5 and Figure S4.

α-Synuclein forms mainly parallel bundles of fibrils when aggregated alone (Figure 5e). The morphology of the aggregates is clearly different when formed in the presence of lipids (Figure 5f, 5g, 5h), as then the fibril bundles have broken apart and form looser tangles. Fewer fibrils lie parallel to one another and there is a significant increase in the number of thinner bundles lying across each other. This effect is significant already at the lowest lipid concentration investigated (L/P = 1). Remarkably, there are no free vesicles present in the surrounding solution at this L/P ratio. At higher lipid contents, the tangled structure of fibrils remains, while excess lipids appear as trapped vesicles in between the fibrils. In the majority of the cases, the vesicles are attached to the fibrils, and in several cases, the vesicles are deformed to non-spherical shape where part of the bilayer wets the fibrillar aggregate. Here, the vesicles adsorb to the fibrils with parts of the bilayer lying parallel to the surface of the fibrillar aggregate, and the bilayer appears planar with little or no curvature. This clearly implies strong interaction between the lipid bilayer and the protein aggregate. The vesicle deformation and wetting of fibrils can be related to the interfacial tension and the bending elasticity modulus in the bilayer [32], and it is more likely to occur for the larger vesicles [33]. Similar behavior has previously been reported for phospholipid vesicles adsorbed to SiO_2_ particles [34], and β2-microglobulin fibrils [7]. Samples with the same composition of lipid and protein as used in the cryo-TEM studies were investigated by means of PT ssNMR. For protein alone, the spectrum is dominated by CP intensity, implying rigid protein structure. No INEPT intensity is visible that does not originate from the buffer. At L/P = 1 molar ratio, the main acyl chain peak (30.5 ppm) is visible among the protein peaks with clearly dominating CP- and DP- over INEPT signals. Increasing L/P to 3.6 (corresponding to a lipid/protein weight ratio of 1/5), the trend is the same but with higher overall intensities from the lipid carbons. At even higher lipid contents (L/P = 18, corresponding to lipid/protein weight ratio 1/1) there is a change in behavior, with the INEPT intensity approaching the DP intensity in the main acyl chain peak (30.5 ppm).

In control cryo-TEM experiments, pre-formed fibrils were incubated with unilamellar vesicles of the same lipid composition DOPC:DOPS 7∶3 (L/P = 3.6) for 2 h before the analysis. The results show that lipids associate also with pre-formed fibril, although the final aggregates differ from those formed in the presence of lipids. The cryo-TEM images (Figure S2) reveal a different morphology compared to co-aggregated samples, and the samples with lipids added to pre-formed fibrils contain bundles similar to those formed in the absence of lipids (Figure 5e). Deformed non-spherical vesicles are attached to the bundles of fibrils. From the PT ssNMR experiments, we conclude that the associated lipids have decreased mobility and that the amount of associated lipids is significantly lower compared to the samples where lipids were present during aggregation.

### Sensitivity to Membrane Composition

**Figure 6:**
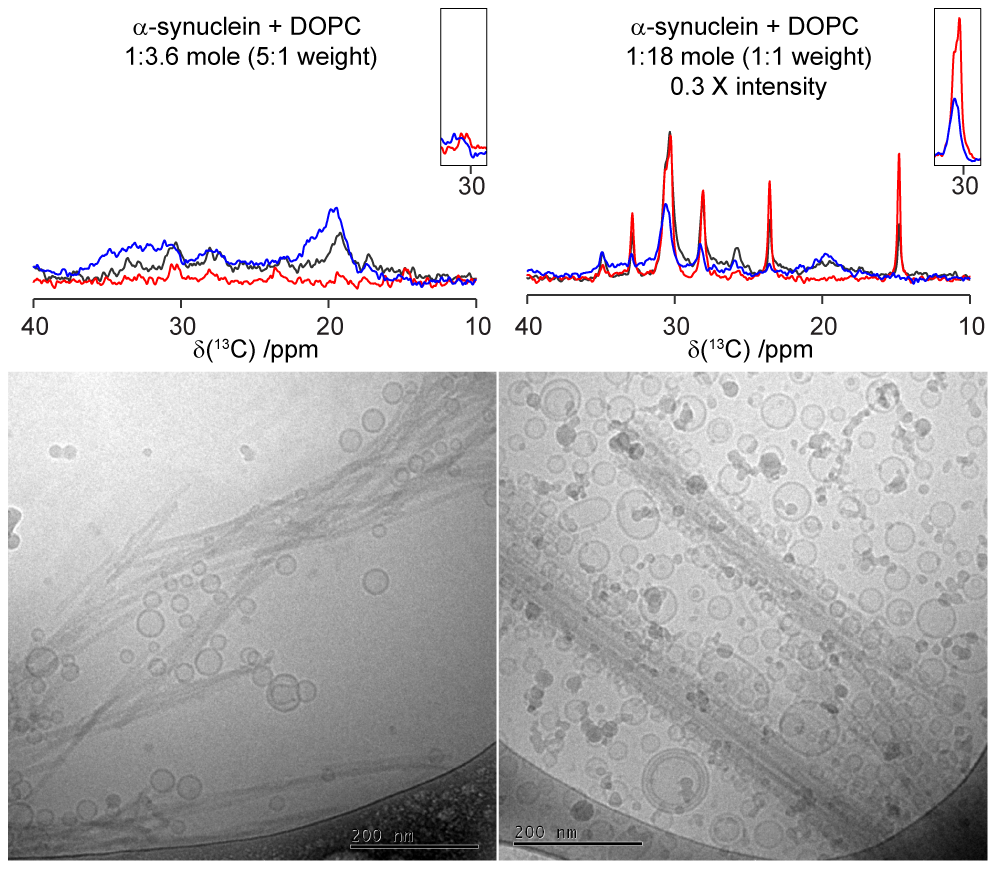
Cryo-TEM images of α-synuclein fibrils formed after co-aggregation with different amounts of DOPC vesicles. Inserted boxed peaks are INEPT and CP signals for the unresolved peaks from C_4–7_ and C_12–15_ adjusted for protein CP signal by baseline correction. Spectra are scaled to give equal protein CP intensity at 20 ppm. Filled dark spots in cryo-TEM images are technique dependent frost defects and are not part of the experimental system. More representative images can be found in Figure S4.

One important finding in this study is that lipid uptake into the co-aggregates is sensitive to lipid composition, and the highest uptake is found for the model membrane that contains anionic lipids. Still, it is clear from the phosphorus analysis in Figure 2 that a small portion of lipids are present also in aggregates formed in the presence of vesicles that contain zwitterionic DOPC only. From the PT ssNMR experiment we again conclude the presence of DOPC in the formed aggregates, although the molecular mobility in these associated lipids is not affected by being co-aggregated with protein and the spectra obtained for DOPC co-aggregated with protein (Figure 2f) is very similar to that of the neat DOPC lamellar phase (Figure 2e) with the same intensity ratios of CP and INEPT for all peaks. Figure 6 shows cryo-TEM images and PT ssNMR spectra for aggregates formed from α-synuclein in the presence of DOPC vesicles for different L/P ratios. The cryo-TEM images clearly show that the amyloid fibrils arrange in bundles in a similar manner to that observed for the fibrils formed in the absence of lipids (Figure 5e). This clearly differs from the tangles of fibrils formed when anionic lipids were present during the aggregation (Figure 5f–h). The fibril bundles remain intact even for the highest lipid concentration. The cryo-TEM images in Figure 6 show the presence of DOPC vesicles in the vicinity of the fibril bundles. However, in contrast to the DOPC/DOPS vesicles present at the tangled fibril aggregates in Figure 5g, 5h, the entrapped DOPC vesicles and do not adsorb and wet to the fibril surface as was observed for the DOPC:DOPS vesicles.

## Discussion

We find here that co-aggregates of protein and lipid form spontaneously in mixtures of α-synuclein and small unilamellar phospholipid vesicles, and that the co-aggregation process is selective with respect to lipid composition. The largest lipid uptake into the amyloid fibrils is found for a model membrane system that contains anionic phosphatidyl serine. This may not seem surprising, as several groups have reported higher affinity for α-synuclein binding to anionic lipid bilayers [6], [8], [9], [35]–[38]. However, here we look at a complementary and different process that is the uptake of lipids into the amyloid aggregates that are the equilibrium structures formed when the monomeric α-synuclein is left to aggregate in the presence of phospholipid vesicles. The PT ssNMR results show that these lipids have higher |S_CH_| and/or have slower dynamics in the co-aggregated state compared to the neat lamellar phase. The saturation limit of lipids in the aggregates was determined from phosphorous analysis to around 9–15 lipids per protein. The PT ssNMR data show the largest decrease in mobility for L/P molar ratios of 1 to 3.6, and cryo-TEM images demonstrate intact but fibril-associated vesicles at L/P = 3.6 and above.

**Figure 7:**
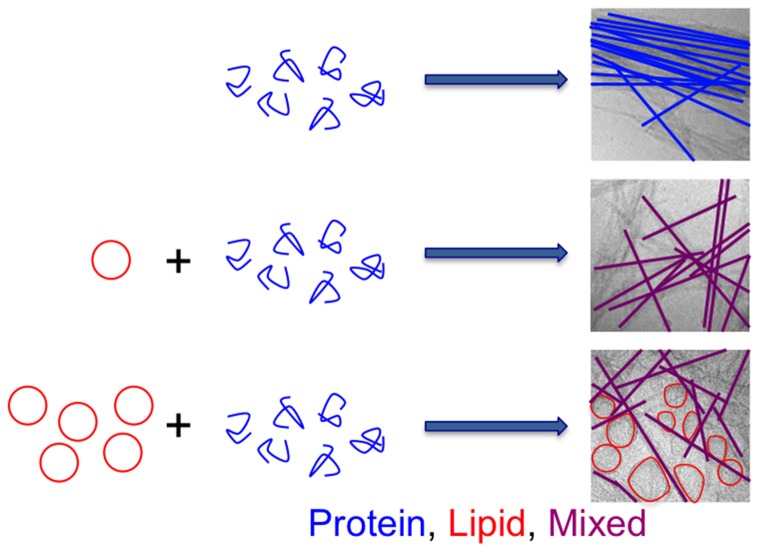
Schematic representation of lipid-protein co-aggregation at different L/P ratios. α-synuclein alone aggregates to fibrils that form bundles. When aggregation takes place in the presence of lipid vesicles (L/P = 1), fibrillar co-aggregates composed of protein and lipids form. These aggregates arrange into mesh-like tangles. At higher L/P ratios, the excess lipid vesicles adsorb to the fibrils, and due to strong interaction with the surface, the vesicles are deformed to non-spherical shape.

The interpretation of the combined data from cryo-TEM, PT ssNMR and phosphorous analysis is illustrated in Figure 7. At lower L/P molar ratios, all available lipids are tightly co-aggregated with fibrils close to saturation, which breaks the fibril bundles and creates the tangled network. No vesicles are observed by cryo-TEM at this L/P ratio, indicating that all lipids are embedded with the protein in co-aggregates. When the lipid concentration is increased to 18 mole per mole protein, excess lipids are trapped as vesicles in the fibril network. This is close to the L/P ratio at which saturation is detected in the quantitative phosphorous assay, suggesting that the trapped vesicles co-sediment with the fibrils. The vesicle are adsorbed to the fibrillar aggregates, and many of them are deformed to non-spherical shape with the bilayer wetting the aggregate surface, implying strong interaction between the lipid bilayer and the protein-lipid co-aggregate. Deformation of vesicles incubated with aggregating α-synuclein has recently also been shown after negative staining by means of electron microscopy [39]. When lipid vesicles are added to pre-formed fibrils, lipids can only access the outer surface of the fibril aggregates, and the bundles therefore remain intact. The combined PT ssNMR and cryo-TEM data then suggest that lipids are adsorbed to the fibrils, leading to reduced mobility, and that the excess intact but deformed lipid vesicles strongly adsorb to the fibrils.

The decreased mobility detected by means of PT ssNMR at L/P molar ratios below 3.6 can originate from different modes of molecular organization. In the following section we discuss some potential scenarios of protein-lipid co-aggregation and how these correlate with the combined results from the PT ssNMR and the cryo-electron micrographs.

Numerous studies have demonstrated adsorption of monomeric α-synuclein to anionic lipid bilayers [6], [8], [9], [35]–[38]. Adsorption of monomeric α-synuclein to the DOPC/DOPS vesicles could potentially lead to a reduced molecular mobility in the phospholipids, which would be detectable in the PT ssNMR experiments. However, the cryo-TEM images in Figure 5f–g show that the fibril bundles are disrupted already at L/P = 1, and for this concentration, no intact lipid vesicles are detected in the whole sample. This implies that the surface properties of the fibrils themselves have been altered by the presence of lipids. Furthermore, the largest reduction in lipid mobility is shown for lower lipid to protein ratios (1–3.6), and an increase in lipid content lead to an overall increased lipid mobility on average. Indeed, the PT ssNMR experiments show that the I_CP_ to I_INEPT_ ratio for all resolved peaks approach that of the neat lamellar phase when the L/P is increased to 18, and for this composition, we observed many associated intact vesicles by means of cryo-TEM. Taken together, this implies that the decrease in mobility in the PT ssNMR experiments originates from lipids associated with the fibrils and not from monomeric or oligomeric protein associated with intact lipid bilayers in vesicles.

We have concluded that lipids co-aggregate with α-synuclein and that lipids are present in the formed aggregate. One can envision a number of different possibilities of the molecular organization in the co-aggregates. Three different scenarios with different lipid structure can be hypothesized to occur individually or in combination:

1. Co-aggregation leads to the formation of a solid (gel) phase or a structure with decreased local lipid dynamics.
2. Lipids form a continuous structure with liquid crystalline phase bilayers or monolayers, interior or exterior in the fibril bundling.
3. Lipids form a discontinuous structure, i.e. isolated lipid molecules that are incorporated into the fibril structure upon co-aggregation.

Scenario 1 implies more ordered lipids with very little INEPT signal. However, we observe significant INEPT signal already at 1∶1 mole of lipid to protein, although the intensity is reduced compared to the neat lipid lamellar phase. A highly ordered solid gel phase can be ruled out based on the absence of a characteristic CP peak at 33–34 ppm originating from acyl chains in an all-trans conformation [40].

Scenario 2 includes the possibilities of monolayer or bilayer coverage of the full fibril or patches of continuous lipid phase. One could also imagine a continuous lipid domain within the interior of the fibril. An adsorbed monolayer, and to less extent a bilayer, at the fibril would experience decreased diffusion rates and thus slower dynamics. This scenario is thus consistent with the observed increase in I_CP_:I_INEPT_ ratio. However, if the lipid dynamics is in the fast regime also after the adsorption, the increase in the I_CP_:I_INEPT_ ratio can be explained by an increased |S_CH_| in the lipid domain. This could originate from an anisotropic extension of the continuous structure, such as a one-dimensional extension along the fibril axis. The increase in the I_CP_:I_INEPT_ ratio could also originate from an increase in *τ*_c_ due to strong attractive interaction between the lipids and the fibrils. These options are both realistic and they are consistent with the present combined data.

Monomeric α-synuclein is believed to adsorb to bilayers via α-helix formation of the N-terminal half of the protein [41]. Only parts of this membrane-associated region of the protein, that is the region towards the central part of the sequence, are considered to take part in the cross beta structure in the fibril formation [8]. It is therefore possible that the N-terminal part of the protein retains its membrane binding properties in the fibril state and thereby stabilizes a continuous bilayer along the fibril axis. The lipid layer could be interior or exterior in the fibril bundling. This interpretation is consistent with recent ssNMR data on protein structure in α-synuclein aggregates, showing that although the major protein fold is not affected by aggregation taking place in the presence of anionic phospholipids, there is a clear influence on the N-terminal part of the protein [39]. The deformed vesicles wetting the surface of fibrils in Figure 5f–h are compatible with an external continued association.

Scenario 3 describes the possibility that individual lipid molecules are incorporated in the fibril and that the lipids do not self-assemble in a continuous structure. This would lead to slower dynamics in the lipid molecules, and this is compatible with the present experimental data. However, the PT ssNMR data from the control experiment where pre-formed fibrils were incubated with lipid vesicles show reduced mobility in lipid hydrocarbon chains, which implies lipid adsorption on the outer accessible surface of the fibrils.

## Conclusion

With support from three complementary techniques, we find co-aggregation of α-synuclein and lipids from anionic model membranes. This is a spontaneous process that occurs if monomeric protein is mixed with lipid vesicles and incubated together. The present data reveal tight internal or external association of lipids with the amyloid fibrils, and the lipids may be incorporated in the fibril structure as isolated molecules, continuous bilayers or monolayers. As a striking complement to the more common view of amyloid protein species associating with membranes, our results open up a new focus on lipid protein co-aggregation, and the observed co-aggregation of protein and lipid provides new insights into the molecular aspects of amyloid aggregation. The results call for new essential approaches in the field of amyloid-lipid interaction, and exploration of its fundamental consequences in living cells.

## Supporting Information

Figure S1 **Thin layer chromatography of phospholipid extracts.** A) Pellet from 36 µM a-synuclein incubated with 900 µM DOPC:DOPS 7∶3. B) Supernatant from 36 µM α-synuclein incubated with 900 µM DOPC:DOPS 7∶3. C) 900 µM DOPC:DOPS 7∶3 supernatant. D) Pellet from 36 µM a-synuclein incubated with 36 µM DOPC:DOPS 7∶3. All samples were incubated at 200 rpm shaking, 37°C, 16 h and then centrifuged after incubation at 13000×g, 5 minutes. Sample C did not produce any pellet and sample D had too low lipid concentration in the supernatant to be detectable. All samples were lyophilized for three days after centrifugation and then dissolved in chloroform:methanol 2∶1. Spotted amounts were as follows: A) 20 µl 1.56× concentrated (calculated on total incubated volume) B) 5 µl 1.56× concentrated C) 10 µl 1.44× diluted D) 10 µl 11.6× concentrated. Stationary phase: Aluminium supported silicagel 60F_254_. Mobile phase: chloroform/methanol/water 65∶25:4 (by volume). Detection: Molybdenum Blue spray reagent.
(TIF)

Figure S2 **Main acyl chain region of the PT ssNMR spectra (top) and cryo-TEM images (bottom, scale bar 200 nm) of preformed α-synuclein fibrils added DOPC:DOPS 7∶3 vesicles.** Filled dark spots in the cryo-TEM image are frost defects and are not part of the experimental system.
(TIF)

Figure S3 **cryo-TEM images, scale bars 200 nm) of α-synuclein fibrils co-aggregated with different amounts of DOPC:DOPS 7∶3 vesicles.** Images are replicates of the images presented in Figure 5. Filled dark spots in cryo-TEM images are technique dependent frost defects and are not part of the experimental system.
(PDF)

Figure S4 **cryo-TEM images, scale bars 200 nm) of α-synuclein fibrils co-aggregated with different amounts of DOPC vesicles.** Images are replicates of the images presented in Figure 6. Filled dark spots in cryo-TEM images are technique dependent frost defects and are not part of the experimental system.
(PDF)

Table S1 **Interpretation of INEPT and CP intensity ratios used in the representation presented in** **Figure 4** **.**
(PDF)
