# Supplementary figures and images for: Membrane Lipid Co-Aggregation with α-Synuclein Fibrils
Source: PLoS One. 2013 Oct 11;8(10):e77235. doi: 10.1371/journal.pone.0077235 (PMC3795653; doi:10.1371/journal.pone.0077235)

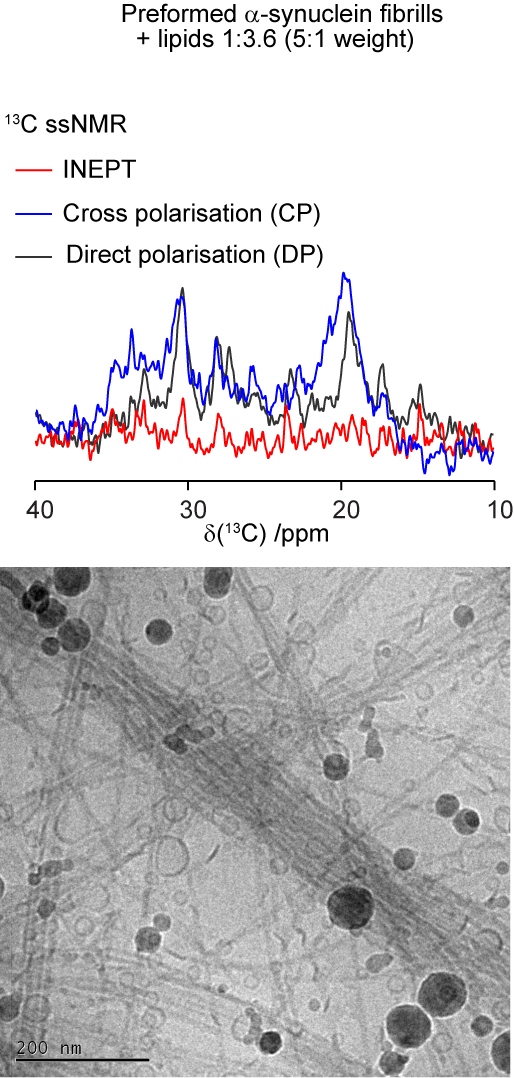

Supplement: Figure S1 — Thin layer chromatography of phospholipid extracts. A) Pellet from 36 µM a-synuclein incubated with 900 µM DOPC:DOPS 7∶3. B) Supernatant from 36 µM α-synuclein incubated with 900 µM DOPC:DOPS 7∶3. C) 900 µM DOPC:DOPS 7∶3 supernatant. D) Pellet from 36 µM a-synuclein incubated with 36 µM DOPC:DOPS 7∶3. All samples were incubated at 200 rpm shaking, 37°C, 16 h and then centrifuged after incubation at 13000×g, 5 minutes. Sample C did not produce any pellet and sample D had too low lipid concentration in the supernatant to be detectable. All samples were lyophilized for three days after centrifugation and then dissolved in chloroform:methanol 2∶1. Spotted amounts were as follows: A) 20 µl 1.56× concentrated (calculated on total incubated volume) B) 5 µl 1.56× concentrated C) 10 µl 1.44× diluted D) 10 µl 11.6× concentrated. Stationary phase: Aluminium supported silicagel 60F254. Mobile phase: chloroform/methanol/water 65∶25:4 (by volume). Detection: Molybdenum Blue spray reagent. (TIF) [file pone.0077235.s001.tif]

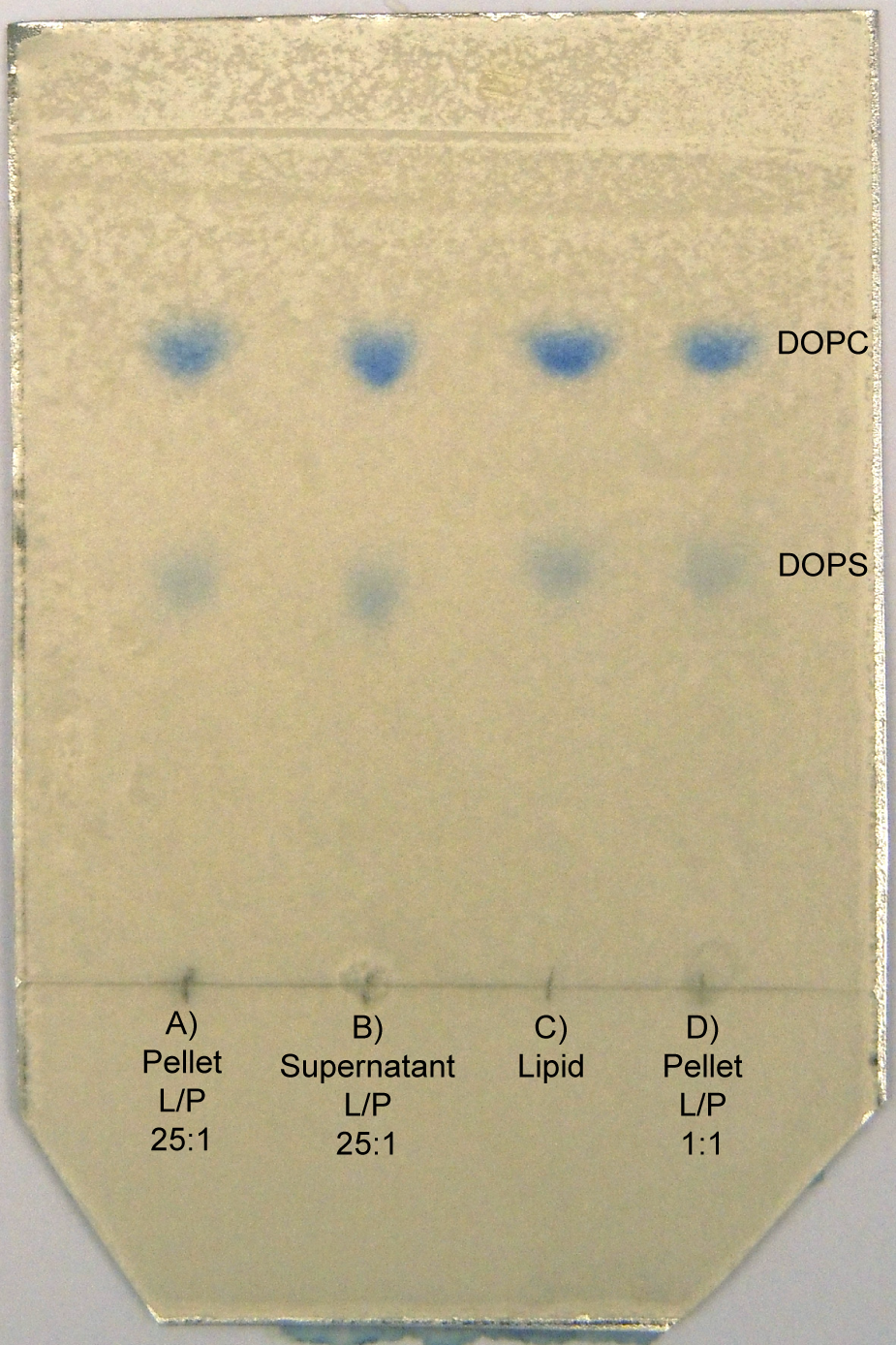

Supplement: Figure S2 — Main acyl chain region of the PT ssNMR spectra (top) and cryo-TEM images (bottom, scale bar 200 nm) of preformed α-synuclein fibrils added DOPC:DOPS 7∶3 vesicles. Filled dark spots in the cryo-TEM image are frost defects and are not part of the experimental system. (TIF) [file pone.0077235.s002.tif]

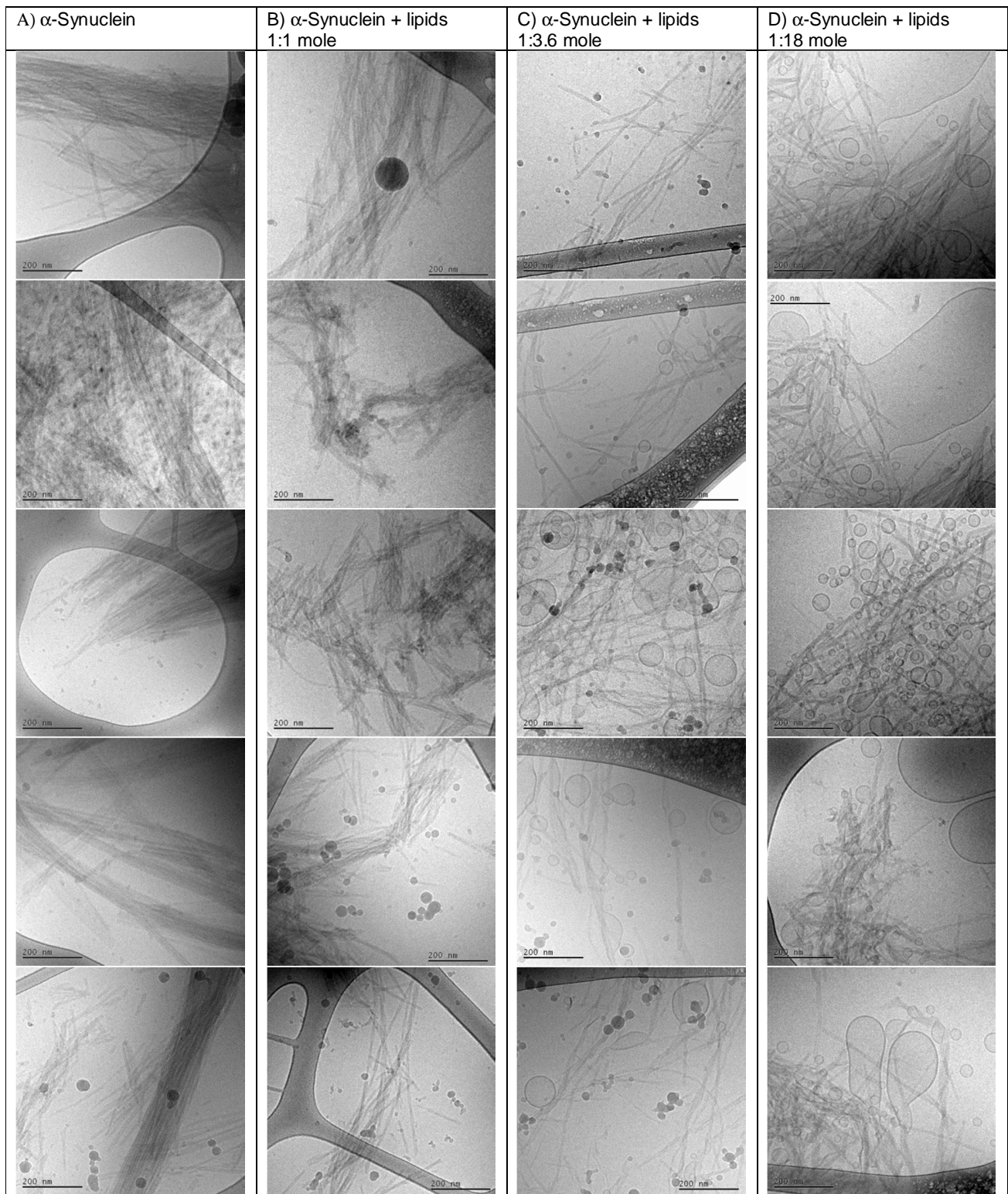

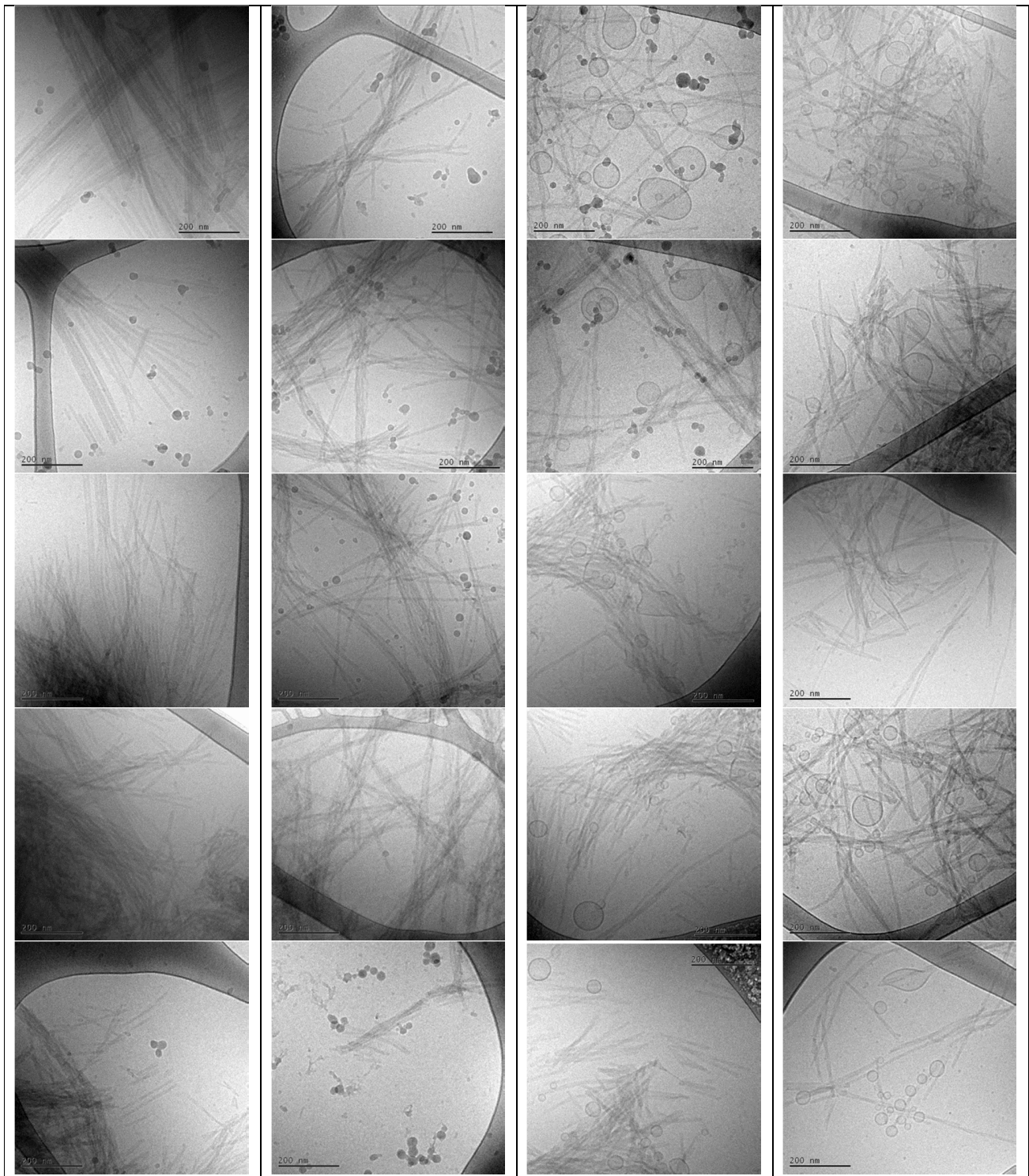

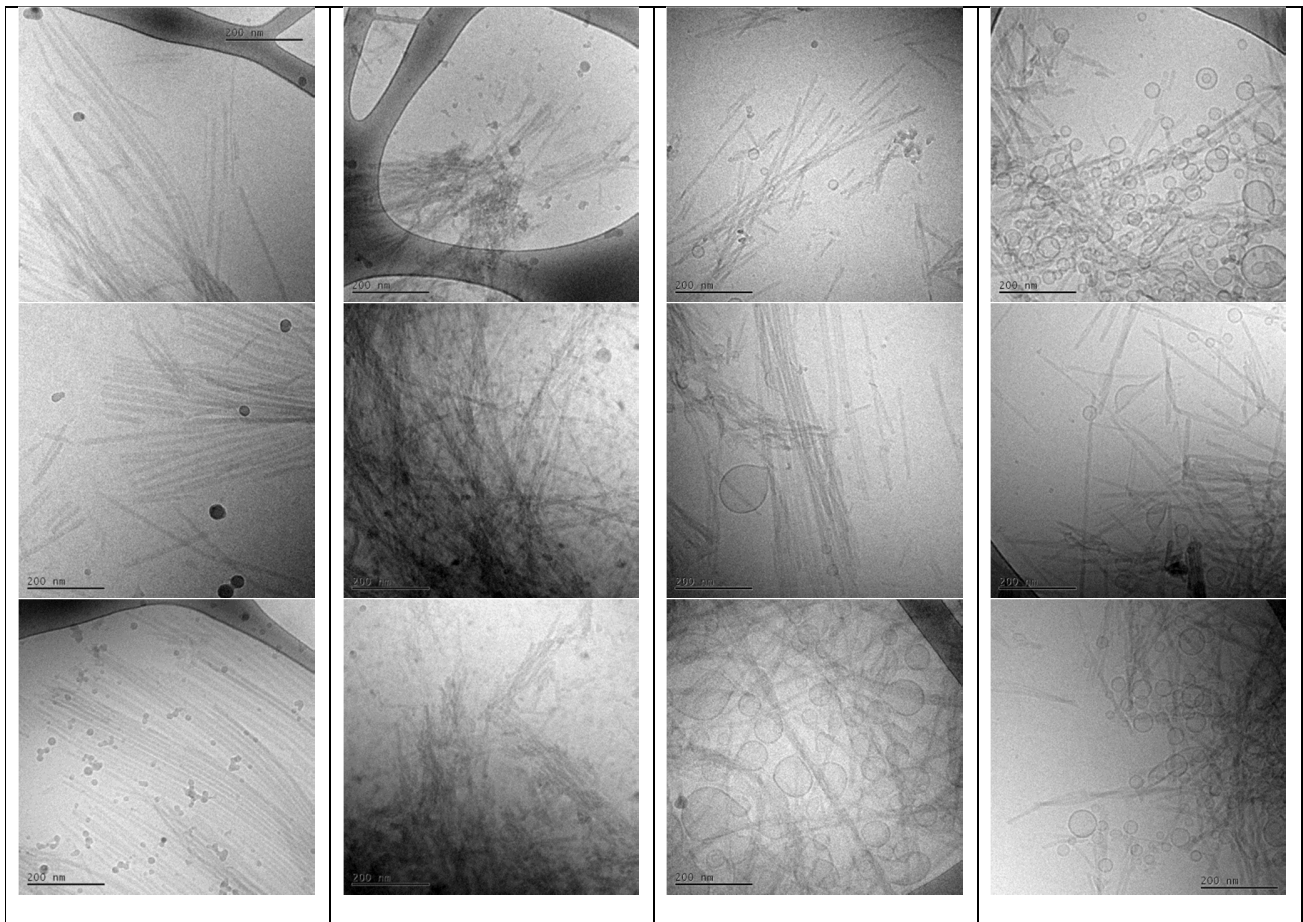

Supplement: Figure S3 — cryo-TEM images, scale bars 200 nm) of α-synuclein fibrils co-aggregated with different amounts of DOPC:DOPS 7∶3 vesicles. Images are replicates of the images presented in Figure 5. Filled dark spots in cryo-TEM images are technique dependent frost defects and are not part of the experimental system. (PDF) [file pone.0077235.s003.pdf]

A)  $\alpha$ -Synuclein + DOPC  
1:3.6 mole

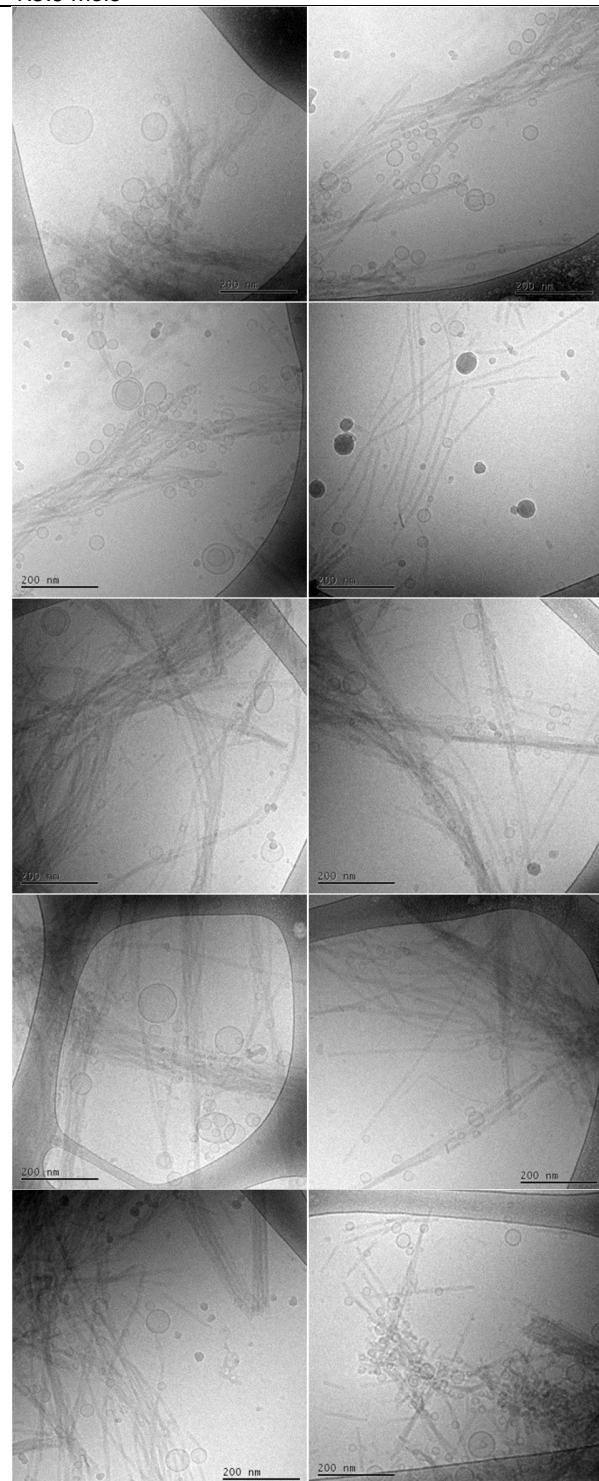

B)  $\alpha$ -Synuclein + DOPC  
1:18 mole

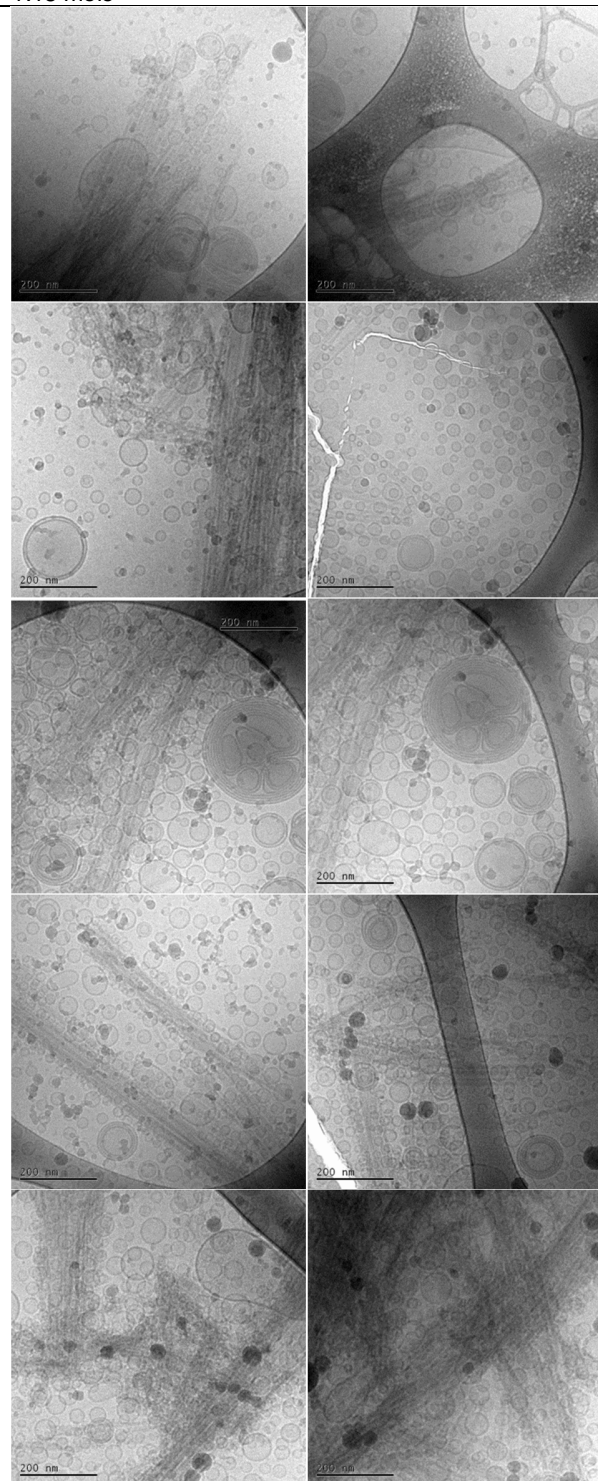

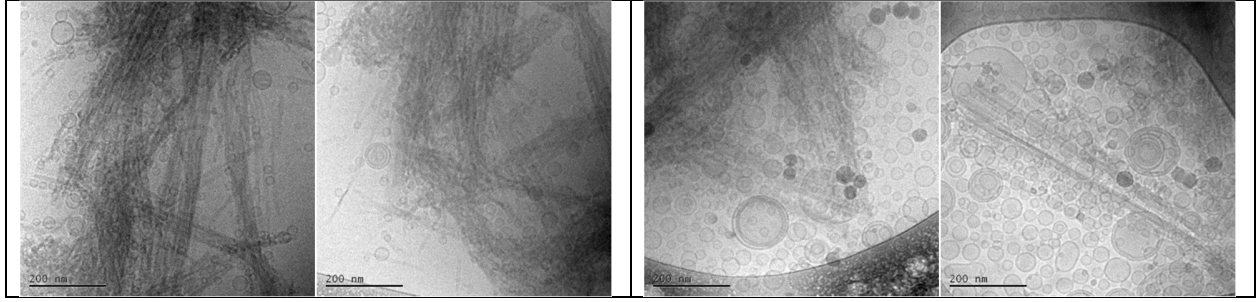

Supplement: Figure S4 — cryo-TEM images, scale bars 200 nm) of α-synuclein fibrils co-aggregated with different amounts of DOPC vesicles. Images are replicates of the images presented in Figure 6. Filled dark spots in cryo-TEM images are technique dependent frost defects and are not part of the experimental system. (PDF) [file pone.0077235.s004.pdf]
