# Supplementary material for: Membrane Lipid Co-Aggregation with α-Synuclein Fibrils
Source: PLoS One. 2013 Oct 11;8(10):e77235. doi: 10.1371/journal.pone.0077235 (PMC3795653; doi:10.1371/journal.pone.0077235)
Supplement: Table S1 — Interpretation of INEPT and CP intensity ratios used in the representation presented in Figure 4 . (PDF) [file pone.0077235.s005.pdf]

| Index | Interpretation |
|---|---|
| A | INEPT>>CP ≈ 0 |
| B | INEPT>4xCP |
| C | INEPT > 2X CP |
| D | INEPT>CP |
| E | INEPT=CP |
| F | CP>INEPT |
| G | CP> 2X INEPT |
| H | CP>4X INEPT |
| I | CP>>INEPT ≈ 0 |

| Carbon | Free lipid | Co-aggregated lipid |
|---|---|---|
| G1 | D | |
| G2 | E | |
| G3 | F | |
| PC-α | C | |
| PC-β | B | |
| PC-γ | *A* | *B* |
| PS-α | C | |
| PS-β | C | |
| 2 | *E* | *I* |
| 3 | *E* | *I* |
| 4-7 | *D* | *F* |
| 8 | *E* | *H* |
| 9 | *C* | *E* |
| 10 | *B* | *C* |
| 11 | *B* | *B* |
| 12-15 | *D* | *F* |
| 16 | *C* | *C* |
| 17 | *B* | *B* |
| 18 | *A* | *A* |
